# Exercise and Caloric Restriction Exert Different Benefits on Skeletal Muscle Metabolism in Aging Condition

**DOI:** 10.3390/nu15235004

**Published:** 2023-12-03

**Authors:** Chanisa Thonusin, Patcharapong Pantiya, Aphisek Kongkaew, Wichwara Nawara, Busarin Arunsak, Sirawit Sriwichaiin, Nipon Chattipakorn, Siriporn C. Chattipakorn

**Affiliations:** 1 Cardiac Electrophysiology Unit, Department of Physiology, Faculty of Medicine, Chiang Mai University, Chiang Mai 50200, Thailand; chanisa.t@cmu.ac.th (C.T.); patcharapong_pantiya@cmu.ac.th (P.P.); sirawit.sriwichaiin@cmu.ac.th (S.S.); nipon.chat@cmu.ac.th (N.C.); 2 Cardiac Electrophysiology Research and Training Center, Faculty of Medicine, Chiang Mai University, Chiang Mai 50200, Thailand; wichwara.nawa@cmu.ac.th (W.N.); busarin.a@cmu.ac.th (B.A.); 3 Center of Excellence in Cardiac Electrophysiology Research, Chiang Mai University, Chiang Mai 50200, Thailand; 4 Research Administration Section, Faculty of Medicine, Chiang Mai University, Chiang Mai 50200, Thailand; aphisek.k@cmu.ac.th; 5 Department of Oral Biology and Diagnostic Sciences, Faculty of Dentistry, Chiang Mai University, Chiang Mai 50200, Thailand

**Keywords:** skeletal muscle, aging, caloric restriction, exercise, metabolism

## Abstract

Exercise and caloric restriction improve skeletal muscle metabolism. However, the benefits of exercise and caloric restriction on skeletal muscle metabolism in aging have never been compared. Seven-week-old male Wistar rats (*n* = 24) were divided into 4 groups (*n* = 6 per group) to receive either normal saline solution for 28 weeks, 150 mg/kg/day of D-galactose for 28 weeks to induce premature aging, 150 mg/kg/day of D-galactose for 28 weeks plus exercise for 16 weeks (week 13–28), or 150 mg/kg/day of D-galactose for 28 weeks plus 30% caloric restriction for 16 weeks (week 13–28). The 17-month-old rats (*n* = 6) were also injected with normal saline solution for 28 weeks as the naturally aged controls. At the end of week 28, total walking distance and fatty acid and carbohydrate oxidation during physical activity were determined. Then, all rats were euthanized for the collection of blood and tibialis anterior muscle. The results showed that D-galactose successfully mimicked the natural aging of skeletal muscle. Exercise and caloric restriction equally improved carbohydrate oxidation during physical activity and myogenesis. However, exercise was superior to caloric restriction in terms of improving fatty acid oxidation and oxidative phosphorylation. Interestingly, caloric restriction decreased oxidative stress, whereas exercise increased oxidative stress of skeletal muscle. All of these findings indicated that the benefits of exercise and caloric restriction on skeletal muscle metabolism during aging were different, and therefore the combination of exercise and caloric restriction might provide greater efficacy in ameliorating skeletal muscle aging.

## 1. Introduction

People are now living longer; therefore, the aging population has been increasing worldwide. It has been estimated that the number of people who are older than 60 years will nearly double from 2002 to 2030 [1]. It is well established that aging is associated with a variety of chronic disorders and organ malfunctions [2]. Skeletal muscle is one of the major organs affected by aging [3]. In other words, aging is a major contributor to skeletal muscle dysfunction [3]. Skeletal muscle function can be classified as both mechanical and metabolic [4]. The mechanical function of skeletal muscle is to maintain posture and produce movement [4]. Importantly, normal protein metabolism in skeletal muscle is required for the effective mechanical function of skeletal muscle [5]. In terms of metabolic function, skeletal muscle is the major contributor to basal energy expenditure as it is a major organ responsible for nutrient utilization and homeostasis [4,6]. To ensure energy supply, skeletal muscle has a high capacity to shift its fuel source between fat and glucose during a fasting and postprandial state, which is known as “metabolic flexibility” [7]. Consequently, skeletal muscle is widely recognized as being the largest insulin-sensitive organ [8]. For this reason, the impairment of metabolic flexibility in skeletal muscle is a cause of metabolic syndrome, which is a major risk factor for atherosclerotic disease [8]. Myogenesis is a potential mechanism responsible for the maintenance of skeletal muscle function [9,10]. A variety of growth factors play an important role in myogenesis, including fibroblast growth factor (FGF), insulin-like growth factor-I (IGF-I), platelet-derived growth factor (PDGF), and vascular endothelial growth factor (VEGF) [11,12]. These growth factors are activated via the phosphoinositide 3-kinase (PI3K)/protein kinase B (AKT) signaling pathway [13].

As previously mentioned, skeletal muscle metabolism is essential for the maintenance of both mechanical and metabolic functions of skeletal muscle. Hence, an improvement in skeletal muscle metabolism during the aging process can contribute to an improvement in skeletal muscle function. It is well known that exercise and caloric restriction improve skeletal muscle metabolism [14,15]. In the aging condition, it was demonstrated that exercise maintained insulin sensitivity in the skeletal muscle of the obese elderly [16,17]. Moreover, exercise maintained fatty acid oxidation enzyme activities, oxidative phosphorylation, and adenosine triphosphate (ATP) production in the skeletal muscle of the elderly [18,19,20]. It was also shown that exercise preserved skeletal muscle regenerative capacity in older individuals [21]. However, the direct effect of exercise on protein metabolism in skeletal muscle in the elderly has not yet been investigated. With regard to caloric restriction, it was shown that this intervention decreased insulin resistance, decreased lipid accumulation, and improved oxidative phosphorylation in the skeletal muscle of aging rodents [22,23]. Nevertheless, studies comparing the beneficial effects of exercise versus caloric restriction on skeletal metabolism in the aging process remain limited.

Several studies demonstrated that injections of D-galactose could be used to create a premature aging model in animals [24,25,26]. Therefore, the objective of this study is to directly compare the potential of exercise versus caloric restriction to improve skeletal muscle metabolism in D-galactose-induced aged rats. Since skeletal metabolism affects the mechanical function of skeletal muscle, the comparative effects of exercise and caloric restriction on locomotor activity were also determined.

## 2. Materials and Methods

### 2.1. Experimental Protocol

The experiments were approved by the Laboratory Animal Center, Chiang Mai University, Chiang Mai, Thailand (approval numbers 2564/RT-0005), in accordance with the Animal Research: Reporting of In Vivo Experiments (ARRIVE) guidelines and the ethical principles of the Association for Assessment and Accreditation of Laboratory Animal Care International (AAALAC). All procedures except euthanasia were conducted at the Laboratory Animal Center, Chiang Mai University, Chiang Mai, Thailand. Euthanasia was performed at the Cardiac Electrophysiology Research and Training Center, Chiang Mai University, Chiang Mai, Thailand. The number of animals per group was calculated using G*Power version 3.1.

Seven-week-old male Wistar rats (*n* = 24) and 17-month-old male Wistar rats (*n* = 6) were included in this study. The 7-week-old rats were randomly divided into 4 groups (*n* = 6 per group) to receive the following by subcutaneous injection: (1) 1 mL/day of normal saline solution (NSS) as a vehicle for 28 weeks (young adult group with sedentary lifestyle); (2) 150 mg/kg/day of D-galactose for 28 weeks (D-gal group) to induce premature aging; (3) 150 mg/kg/day of D-galactose for 28 weeks and an exercise regime for 16 weeks (at week 13–28; D-gal with EX group); and (4) 150 mg/kg/day of D-galactose for 28 weeks and 30% caloric restriction for 16 weeks (at week 13–28; D-gal with CR group). The injections were given for 28 weeks; therefore, these rats received the interventions until they reached 35 weeks of age. The 17-month-old rats were also subcutaneously injected with 1 mL/day of NSS for 28 weeks, i.e., until the rats reached 24 months of age (natural aging group).

**Figure 1:**
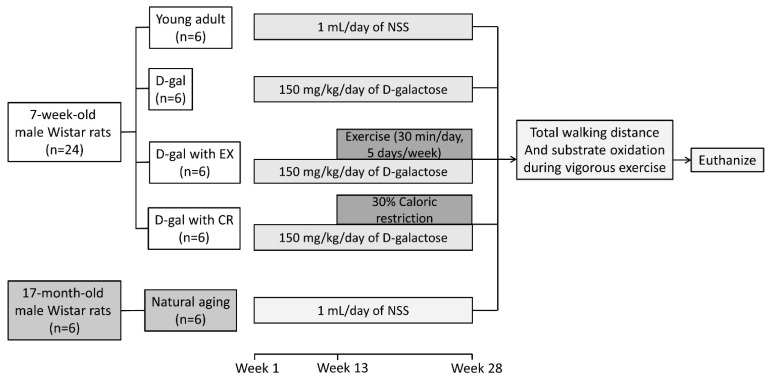
The experimental protocol. D-gal = D-galactose-induced premature aging, D-gal with EX = D-gal with exercise, D-gal with CR = D-gal with caloric restriction.

At the end of week 28, total walking distance and whole-body fatty acid and carbohydrate oxidation during physical activity were determined. Then, all rats were euthanized after 5 h of fasting, and blood and tibialis anterior muscle tissue samples were collected. Euthanasia was performed under 100% isoflurane inhalation, followed by decapitation. The experimental protocol is summarized in Figure 1.

### 2.2. Exercise Training Protocol

The exercise regime was performed on a rodent treadmill (Panlab/Harvard Instruments, Barcelona, Spain) as described in a previous study [27]. Briefly, the treadmill was set at 0° inclination with an initial speed of 8 cm/s and a step up of 2 cm/s per day until it reached 20 cm/s. The exercise duration per day was half an hour with a frequency of 5 days/week [28,29].

### 2.3. Blood Metabolic Parameters

The measurement of blood metabolic parameters, including insulin sensitivity profile and lipid profile, was detailed in a prior study [27]. In brief, fasting plasma glucose was determined using a colorimetric assay kit (ERBA Mannheim, Mannheim, Germany). Insulin level was measured using a sandwich enzyme-linked immunosorbent assay (ELISA) kit (LINCO Research, Saint Charles, MO, USA). To identify the degree of insulin resistance, the homeostatic model assessment of insulin resistance (HOMA-IR) was calculated. Triglyceride and total cholesterol levels were evaluated using colorimetric assay kits (ERBA Mannheim, Mannheim, Germany). Plasma HDL level was analyzed using a commercial assay kit (Biovision, Inc., Milpitas, CA, USA). Finally, the LDL level was estimated using the Friedewald equation [30].

### 2.4. Serum Testosterone Level

Serum testosterone level was measured using electrochemiluminescence immunoassay (ECLIA) with a Cobas e601 automated analyzer (Roche Diagnostics, Basel, Switzerland).

### 2.5. Walking Distance to Determine Locomotor Activity

Each rat was placed in the center of a circular-based box, 70 cm in diameter and 50 cm high, and allowed to freely walk for 10 min. A video camera was turned on to record all testing sessions. Total walking distance within a 10 min duration was calculated using SMART Video Tracking System version 3.0 (Panlab/Harvard Instruments, Barcelona, Spain).

### 2.6. Whole-Body Fatty Acid and Carbohydrate Oxidation during Physical Activity

Whole-body fatty acid and carbohydrate oxidation during physical activity were determined by indirect calorimetry during running on a speed-ramped treadmill, as detailed in a prior study [27]. Briefly, a closed-system treadmill with a gas analyzer (the OxyletPro, Panlab/Harvard Instruments, Barcelona, Spain) was set at an inclination of 15° with an initial speed of 16 cm/s. The speed was automatically increased at a rate of 2 cm/s every 2 min. The oxygen uptake (VO_2_) and the volume of exhaled carbon dioxide (VCO_2_) were recorded every 1 s using metabolism software version 3.0 (Panlab/Harvard Instruments, Barcelona, Spain). The procedure was stopped when the rat experienced exhaustion, as indicated by at least 3 times of a 2 s drop-down period. Whole-body carbohydrate and fatty acid oxidation rates during physical activity were calculated using VO_2_ and VCO_2_ at 75% of VO_2_max [31,32,33].

### 2.7. Skeletal Muscle Metabolomics

To extract skeletal muscle metabolomes, 40 mg of pulverized skeletal muscle tissue was added to 1 mL of high-performance liquid chromatography (HPLC)-graded methanol:water:chloroform (7:2:1). The mixture was immediately sonicated on ice for 30 s and allowed to rest on ice for 5 min. After that, the mixture was centrifuged at 15,300 rpm at 4 °C for 10 min. The supernatant was then transferred to an autosampler vial.

The liquid chromatography coupled with mass spectrometry (LC/MS) instrument used in this study was a 1260 infinity II LC/6546 quadrupole time-of-flight (Q-TOF) MS (Agilent Technologies, Santa Clara, CA, USA). The MS parameters used were detailed in a previous study [34]. Regarding the chromatographic conditions, amino acids, palmitic acid, oleic acid, glycolysis metabolomes, Krebs cycle metabolomes, and ATP were measured under hydrophilic interaction liquid chromatography (HILIC) in the negative ion mode, while acylcarnitine intermediates of palmitic acid and oleic acid were measured under reversed-phase liquid chromatography (RPLC) in the positive ion mode. The details of both chromatographic conditions are described in a previous study [35]. The injection volume per sample for each chromatographic condition was 10 µL.

The peak area of each metabolome was quantified using MassHunter Quantitative Analysis Software version 10.1 (Agilent Technologies, Santa Clara, CA, USA) and was then normalized using MetaboDrift, as detailed in prior studies [34,35].

### 2.8. Skeletal Muscle Protein Expression Analyses

Expression of skeletal muscle proteins was determined using Western blot analysis. All steps of the Western blot are described in a previous study [36]. In brief, skeletal muscle tissue was homogenized with a lysis buffer containing 1% Nonidet P-40, 0.5% sodium deoxycholate, 0.1% SDS in 1× PBS, and 1× protease inhibitor cocktail (Merck, KGaA, Darmstadt, Germany). After incubation on ice for 30 min, the homogenate was centrifuged at 13,000 rpm for 10 min to collect the protein. Then, 2 mg/mL of protein was mixed with the loading buffer containing 5% mercaptoethanol, 0.05% bromophenol blue, 75 nM Tris-HCl, 2% SDS, and 10% glycerol with pH 6.8 in a 4:1 proportion. The mixture was heated at 95 °C for 10 min and loaded into sodium dodecyl sulfate (SDS)-polyacrylamide gel electrophoresis. Thereafter, the protein was transferred to 0.45 μm pore size nitrocellulose membranes (GE Healthcare Bio-Sciences, Marlborough, MA, USA) in a glycine/methanol-transfer buffer using a Wet/Tank blotting system (Bio-Rad Laboratories, Hercules, CA, USA). Membranes were blocked in 5% bovine serum albumin for 1 h. To detect the levels of protein expression, the membranes were then incubated overnight at 4 °C with primary antibodies. After washing the primary antibodies, secondary antibodies were added for 1 h at room temperature. The membranes were exposed to an ECL Western blotting substrate (Bio-Rad Laboratories, Hercules, CA, USA), and a densitometric analysis was performed using the ChemiDoc Touch Imaging system (Bio-Rad Laboratories, Hercules, CA, USA), followed by ImageJ version 1.52a (NIH, Bethesda, MD, USA). In each sample, the expression level of each specific protein was normalized by a total protein level obtained from Ponceau S staining.

### 2.9. Skeletal Muscle Reactive Oxygen Species (ROS) Level

One hundred µg/mL of skeletal muscle protein was incubated with dichloro-dihydro-fluorescein diacetate dye (2 µM) at 25 °C for 20 min. Fluorescence intensity was measured using a fluorescent microplate reader (Bio-Tek Instruments Inc., Winooski, VT, USA) with excitation and emission wavelengths of 485 nm and 530 nm, respectively. A high level of fluorescent intensity indicated a high level of ROS [37].

### 2.10. Statistical Analyses

All data are reported as the mean ± standard error of the mean (SEM). A one-way analysis of variance (ANOVA) followed by a Tukey test was used for the comparison between groups. Statistical significance was defined as a *p*-value < 0.05.

## 3. Results

### 3.1. Exercise and Caloric Restriction Equally Decreased Body Weight and Visceral Fat Weight in D-Galactose-Induced Aged Rats

**Figure 2:**
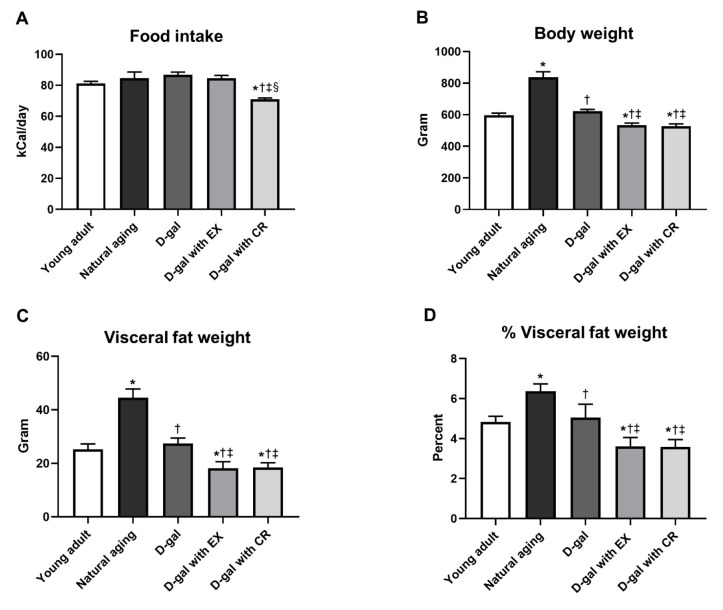
Average food intake from week 1 to 28 (**A**), body weight (**B**), visceral fat weight (**C**), and % visceral fat weight (**D**). Data are reported as mean ± SEM. *n* = 6 per group; D-gal = D-galactose-induced premature aging, D-gal with EX = D-gal with exercise, D-gal with CR = D-gal with caloric restriction; * *p* < 0.05 when compared to young adult, † *p* < 0.05 when compared to natural aging, ‡ *p* < 0.05 when compared to D-gal, § *p* < 0.05 when compared to D-gal with EX.

As expected, D-galactose-treated rats with caloric restriction consumed the smallest amount of food, whereas food intake among the other five groups of rats did not differ (Figure 2A). Interestingly, naturally aged rats had higher body and visceral fat weights than those of young adults and D-galactose-induced aged rats (Figure 2B–D). Exercise and caloric restriction resulted in an equal reduction in body weight and visceral fat weight in D-galactose-induced aged rats (Figure 2B–D).

### 3.2. Caloric Restriction Is Superior to Exercise in Terms of Improving Systemic Insulin Sensitivity in D-Galactose-Induced Aged Rats

**Figure 3:**
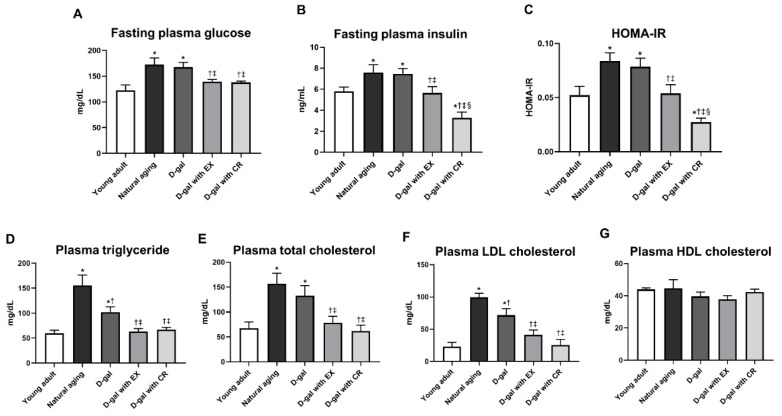
Blood metabolic parameters: fasting plasma glucose (**A**), fasting plasma insulin (**B**), HOMA-IR (**C**), plasma triglycerides (**D**), plasma total cholesterol (**E**), plasma LDL cholesterol (**F**), and plasma HDL cholesterol (**G**). Data are reported as mean ± SEM. *n* = 6 per group; D-gal = D-galactose-induced premature aging, D-gal with EX = D-gal with exercise, D-gal with CR = D-gal with caloric restriction; * *p* < 0.05 when compared to young adult, † *p* < 0.05 when compared to natural aging, ‡ *p* < 0.05 when compared to D-gal, § *p* < 0.05 when compared to D-gal with EX.

Rats who underwent natural aging and D-galactose-induced aging had higher systemic insulin resistance than those of the young adult rats. This was indicated by increased levels of fasting plasma glucose, plasma insulin, and HOMA-IR (Figure 3A–C). D-galactose-induced aged rats with exercise showed a decrease in the levels of those parameters to become comparable with those of the young adults (Figure 3A–C). Interestingly, the levels of fasting plasma insulin and HOMA-IR were significantly lower in D-galactose-induced aged rats with caloric restriction when compared to those of the young adults (Figure 3A–C). All of these results suggest that the effectiveness of caloric restriction on the improvement of systemic insulin sensitivity in aging conditions is greater than that of exercise. 

### 3.3. Exercise and Caloric Restriction Equally Improve the Blood Lipid Profile in D-Galactose-Induced Aged Rats

Naturally aged and D-galactose-induced aged rats had higher levels of plasma triglycerides, total cholesterol, and LDL cholesterol than those of the young adults (Figure 3D–F), while plasma HDL cholesterol levels were no different among all six groups (Figure 3G). Interestingly, the levels of plasma triglyceride and LDL cholesterol were significantly greater in naturally aged rats when compared with those of D-galactose-induced aged rats (Figure 3D,F). Exercise and caloric restriction resulted in equally decreased plasma triglyceride, total cholesterol, and LDL cholesterol levels in D-galactose-induced aged rats, and the levels became comparable with those of the young adults (Figure 3D–F). 

### 3.4. D-Galactose Administration Results in Cellular Senescence of Skeletal Muscle Which Cannot Be Attenuated by Either Exercise or Caloric Restriction

**Figure 4:**
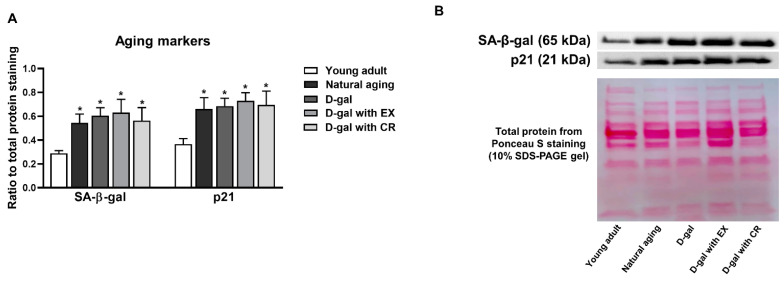
Expression of aging marker-related proteins in skeletal muscle (**A**,**B**). Data are reported as mean ± SEM. *n* = 6 per group; D-gal = D-galactose-induced premature aging, D-gal with EX = D-gal with exercise, D-gal with CR = D-gal with caloric restriction; * *p* < 0.05 when compared to young adult.

The levels of aging markers, including the senescence-associated proteins β-galactosidase (SA-β-gal) and p21, were evaluated in skeletal muscle (Figure 4A,B). In comparison to the young adults, the results showed that the levels of these proteins were higher in naturally aged and D-galactose-induced aged rats (Figure 4A,B). Interestingly, these protein levels were no different between the naturally aged and D-galactose-induced aged rats (Figure 4A,B), indicating that D-galactose administration successfully mimicked the natural senescence of skeletal muscle. Exercise and caloric restriction failed to ameliorate the cellular senescence of skeletal muscle. This was indicated by no reduction in the expression of the proteins SA-β-gal and p21 in the skeletal muscle of D-galactose-treated rats in the exercise or caloric restriction groups (Figure 4A,B).

### 3.5. Exercise and Caloric Restriction Equally Alleviate the Impairment of Growth Factor Signaling in Skeletal Muscle and Improve Carbohydrate Oxidation during Physical Activity—However, These Interventions Fail to Attenuate Skeletal Muscle Insulin Sensitivity in the Sedentary State in the Aging Condition

**Figure 5:**
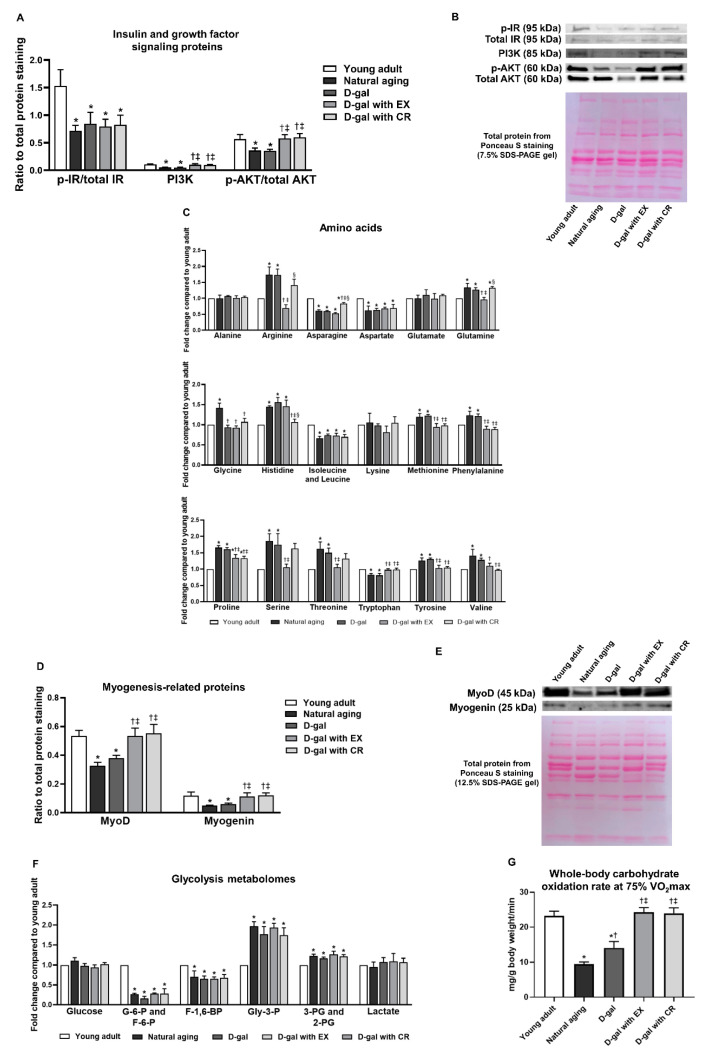
Expression of insulin and growth factor signaling proteins in skeletal muscle (**A**,**B**), levels of amino acids in skeletal muscle (**C**), expression of myogenesis-related proteins in skeletal muscle (**D**,**E**), levels of glycolysis metabolomes in skeletal muscle (**F**), and whole-body carbohydrate oxidation rate at 75% VO_2_max (**G**). Data are reported as mean ± SEM. *n* = 6 per group; D-gal = D-galactose-induced premature aging, D-gal with EX = D-gal with exercise, D-gal with CR = D-gal with caloric restriction; * *p* < 0.05 when compared to young adult, † *p* < 0.05 when compared to natural aging, ‡ *p* < 0.05 when compared to D-gal, § *p* < 0.05 when compared to D-gal with EX; G-6-P = glucose-6-phosphate, F-6-P = fructose-6-phosphate, F-1,6-BP = fructose-1,6-bisphosphate, Gly-3-P = glyceraldehyde-3-phosphate, 2-PG = 2-phosphoglyceric acid, and 3-PG = 3-phosphoglyceric acid.

We measured the levels of proteins involved in insulin and growth factor signaling pathways, including insulin receptor (IR), phosphoinositide 3-kinase (PI3K), and protein kinase B (AKT) (Figure 5A,B). We observed that the expression of the proteins p-IR/total IR, PI3K, and p-AKT/total AKT was lower in the skeletal muscle of naturally aged and D-galactose-induced aged rats when compared to those of young adults (Figure 5A,B). All of these findings indicated aging-induced disruptive insulin and growth factor signaling in skeletal muscle. Interestingly, the expression of the proteins PI3K and p-AKT/total AKT could be restored by exercise and caloric restriction, while the expression of p-IR/total IR proteins was unaltered following both interventions (Figure 5A,B). All of these results suggested that exercise and caloric restriction equally improved skeletal muscle growth factor signaling but failed to improve insulin signaling in the aging condition.

We also evaluated the effects of exercise and caloric restriction on amino acid metabolism and myogenesis in skeletal muscle (Figure 5C–E). The results demonstrated that aging caused impaired amino acid metabolism, as indicated by the alteration of several amino acid levels in the skeletal muscle of naturally aged and D-galactose-induced aged rats (details are shown in Figure 5C and Supplementary Table S1). In addition, naturally aged and D-galactose-induced aged rats exhibited a reduction in the myogenesis-related proteins: Myogenic differentiation 1 (MyoD) and myogenin (Figure 5D,E), all of which indicated the impairment of myogenesis. Consistent with growth factor signaling proteins, exercise and caloric restriction ameliorated the alteration of amino acid levels in skeletal muscle (details are shown in Figure 5C and Supplementary Table S1), as well as restoring the levels of myogenesis-related proteins (Figure 5D,E) in D-galactose-induced aged rats. In other words, exercise and caloric restriction equally alleviated the disruption of growth factor signaling in skeletal muscle.

Consistent with p-IR/total IR proteins, naturally aged and D-galactose-induced aged rats exhibited a change in several glycolysis metabolomes in skeletal muscle, and this could not be improved by either exercise or caloric restriction (details are shown in Figure 5F and Supplementary Table S1). In fact, our results indicated that exercise and caloric restriction failed to attenuate aging-induced skeletal muscle insulin resistance in the sedentary state. In contrast, the impairment of skeletal muscle carbohydrate oxidation during physical activity could be restored by these interventions, as indicated by the restoration of whole-body carbohydrate oxidation rate at 75% VO_2_max in D-galactose-induced aged rats with exercise or caloric restriction (Figure 5G).

### 3.6. Exercise Is More Effective than Caloric Restriction in Improving Skeletal Muscle Fatty Acid Oxidation in the Aging Condition

**Figure 6:**
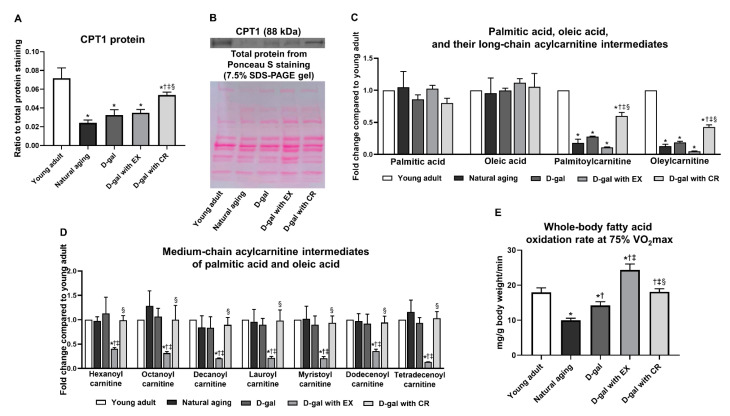
Expression of CPT protein in skeletal muscle (**A**,**B**), levels of palmitic acid, oleic acid, and their long-chain acylcarnitine intermediates in skeletal muscle (**C**), medium-chain acylcarnitine intermediates of palmitic acid and oleic acid in skeletal muscle (**D**), and whole-body fatty acid oxidation rate at 75% VO_2_max (**E**). Data are reported as mean ± SEM. *n* = 6 per group; D-gal = D-galactose-induced premature aging, D-gal with EX = D-gal with exercise, D-gal with CR = D-gal with caloric restriction; * *p* < 0.05 when compared to young adult, † *p* < 0.05 when compared to natural aging, ‡ *p* < 0.05 when compared to D-gal, § *p* < 0.05 when compared to D-gal with EX.

Skeletal muscle fatty acid uptake was determined by the expression of the carnitine palmitoyltransferase 1 (CPT1) protein (Figure 6A,B) and the levels of long-chain acylcarnitine intermediates of palmitic acid (palmitolylcarnitine) and oleic acid (oleylcarnitine) in skeletal muscle (Figure 6C, Supplementary Table S1). The results revealed that aging caused a reduction in skeletal muscle fatty acid uptake, as indicated by lower levels of CPT1 protein, palmitoylcarnitine, and oleylcarnitine in the skeletal muscle of naturally aged and D-galactose-induced aged rats, when compared with those of young adults (Figure 6A–C, Supplementary Table S1). Caloric restriction could partially improve skeletal muscle fatty acid uptake in the aging condition, as shown by increasing CPT protein, palmitoleylcarnitine, and oleylcarnitine levels in D-galactose-treated rats with caloric restriction, but these levels remained lower than those of the young adults (Figure 6A–C, Supplementary Table S1). On the other hand, these changes were not observed in D-galactose-treated rats with exercise (Figure 6A–C, Supplementary Table S1).

Skeletal muscle fatty acid oxidation in the sedentary state was evaluated by the levels of medium-chain acylcarnitine intermediates of palmitic acid and oleic acid in skeletal muscle (Figure 6D, Supplementary Table S1). Although the levels of palmitoylcarnitine and oleylcarnitine in skeletal muscle were lower in naturally aged and D-galactose-induced aged rats, the levels of medium-chain acylcarnitine intermediates of palmitic and oleic acids were no different from those of the young adults (Figure 6D, Supplementary Table S1). These findings suggested that there was an accumulation of intermediate metabolomes of fatty oxidation in the skeletal muscle of aged rats, implying that aging resulted in incomplete fatty acid oxidation of skeletal muscle. Following exercise, the levels of these medium-chain acylcarnitines were significantly decreased (Figure 6D, Supplementary Table S1), indicating an alleviation of aging-induced incomplete fatty acid oxidation in skeletal muscle. Nevertheless, the reductions in these medium-chain acylcarnitines were not exhibited in D-galactose-treated rats with caloric restriction (Figure 6D, Supplementary Table S1).

Skeletal muscle fatty acid oxidation during physical activity was determined by the whole-body fatty acid oxidation rate at 75% VO_2_max. The results showed that aging also caused a reduction in skeletal muscle fatty acid oxidation during physical activity, but this reduction is more severe in naturally aged rats than that of D-galactose-treated rats (Figure 6E). Caloric restriction could restore this change back to the level of young adults (Figure 6E). Interestingly, exercise further increased skeletal muscle fatty acid oxidation during physical activity when compared to that of young adults (Figure 6E).

### 3.7. Exercise Improves Oxidative Phosphorylation and ATP Production of Skeletal Muscle in Aging Condition

**Figure 7:**
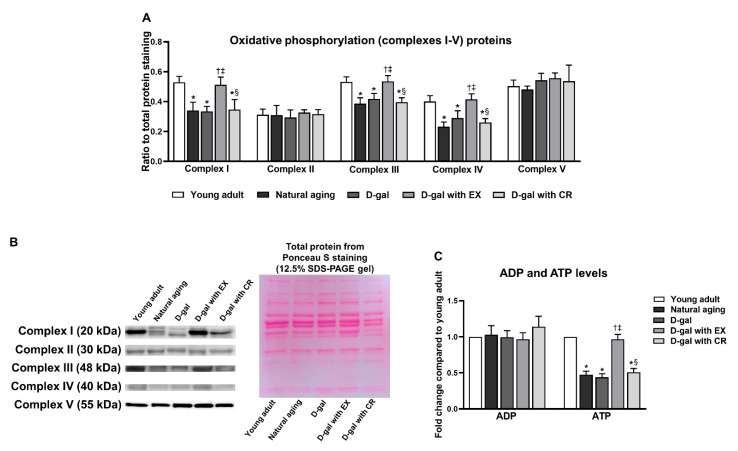
Expression of complexes I–V proteins in skeletal muscle (**A**,**B**), and levels of adenosine diphosphate (ADP) and adenosine triphosphate (ATP) in skeletal muscle (**C**). Data are reported as mean ± SEM. *n* = 6 per group; D-gal = D-galactose-induced premature aging, D-gal with EX = D-gal with exercise, D-gal with CR = D-gal with caloric restriction; * *p* < 0.05 when compared to young adult, † *p* < 0.05 when compared to natural aging, ‡ *p* < 0.05 when compared to D-gal, § *p* < 0.05 when compared to D-gal with EX.

The expression of oxidative phosphorylation-related proteins (complexes I–V) and ATP levels in skeletal muscle were investigated (Figure 7A–C). We observed that aging resulted in disruptive oxidative phosphorylation in skeletal muscle, as indicated by lower levels of complexes I, III, and IV proteins in the skeletal muscle of naturally aged and D-galactose-induced aged rats (Figure 7A,B). In addition, ATP levels were lower in the skeletal muscle of naturally aged and D-galactose-induced aged rats, suggesting aging induced a decrease in skeletal muscle ATP production (Figure 7C). Exercise could restore the levels of complexes I, III, IV proteins, and ATP to be comparable with those of young adults (Figure 7C). Nonetheless, these parameters could not be improved by caloric restriction (Figure 7C).

### 3.8. Caloric Restriction Decreases Aging-Induced Oxidative Stress in Skeletal Muscle, While Exercise Aggravates Aging-Induced Oxidative Stress in Skeletal Muscle

**Figure 8:**
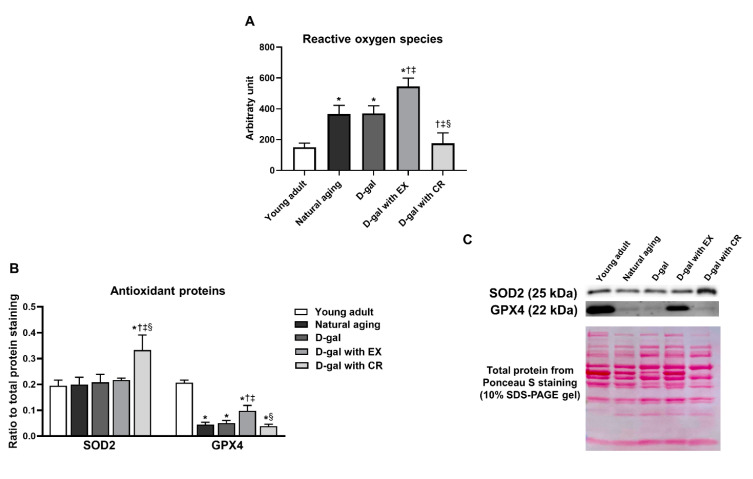
ROS level in skeletal muscle (**A**), and expression of antioxidant proteins in skeletal muscle (**B**,**C**). Data are reported as mean ± SEM. *n* = 6 per group; D-gal = D-galactose-induced premature aging, D-gal with EX = D-gal with exercise, D-gal with CR = D-gal with caloric restriction; * *p* < 0.05 when compared to young adult, † *p* < 0.05 when compared to natural aging, ‡ *p* < 0.05 when compared to D-gal, § *p* < 0.05 when compared to D-gal with EX.

The ROS level in skeletal muscle was higher in the skeletal muscle of naturally aged and D-galactose-induced aged rats than that of young adults, indicating aging-induced increased oxidative stress (Figure 8A). Caloric restriction could restore the level of ROS in skeletal muscle to be comparable with that of young adults (Figure 8A). However, exercise further increased ROS levels in skeletal muscle (Figure 7A).

### 3.9. Caloric Restriction Is Superior to Exercise in Terms of Improving Antioxidative Capacity in Skeletal Muscle in the Aging Condition

Antioxidative capacity was evaluated by the expression of the proteins superoxide dismutase 2 (SOD2) and glutathione peroxidase 4 (GPX4) (Figure 8B,C). We found that the expression of skeletal muscle SOD2 protein was no different between young adult rats, naturally aged rats, and rats treated with D-galactose (Figure 8B,C). Interestingly, caloric restriction significantly increased SOD2 protein in skeletal muscle, while exercise did not change the level of expression of this protein (Figure 8B,C). Unlike SOD2, the expression of the skeletal muscle protein GPX4 was lower in naturally aged and D-galactose-induced aged rats when compared to that of young adults (Figure 8B,C). In D-galactose-induced aged rats, exercise significantly increased the expression of the protein GPX4, but the level remained significantly lower than that of the young adults (Figure 8B,C). However, caloric restriction failed to increase the level of expression of this protein (Figure 8B,C).

### 3.10. Exercise Is Superior to Caloric Restriction in Terms of Improving Locomotor Activity in Aging Condition

**Figure 9:**
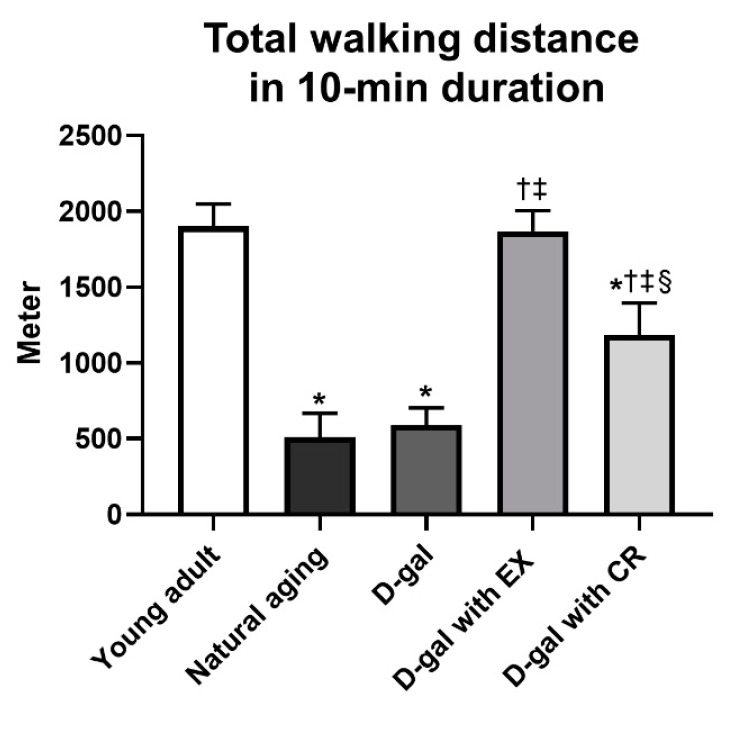
Total walking distance in 10 min duration. Data are reported as mean ± SEM. *n* = 6 per group; D-gal = D-galactose-induced premature aging, D-gal with EX = D-gal with exercise, D-gal with CR = D-gal with caloric restriction; * *p* < 0.05 when compared to young adult, † *p* < 0.05 when compared to natural aging, ‡ *p* < 0.05 when compared to D-gal, § *p* < 0.05 when compared to D-gal with EX.

Locomotor activity was determined by the total walking distance within a 10 min duration (Figure 9). The results revealed that the walking distance of naturally aged and D-galactose-induced aged rats was lower than that of young adults (Figure 9). Both exercise and caloric restriction increased the walking distance, but only exercise restored the distance to become comparable to that of the young adults (Figure 9).

## 4. Discussion

In this study, we used D-galactose to induce aging. To confirm that D-galactose could mimic the natural aging of skeletal muscle, cellular senescence markers in skeletal muscle were compared between naturally aged rats and D-galactose-treated rats. These markers were SA-β-gal and p21. The biomarker p21 mediates G1 growth arrest, resulting in the termination of the cell cycle [38]. SA-β-gal activity is usually increased in the organs of older people and animals [39]. When compared with the young adults, we found that both naturally aged and D-galactose-treated rats had increased expression of the proteins p21 and SA-β-gal in skeletal muscle, with no significant difference between these two groups. All of these findings indicated that D-galactose administration successfully mimicked the natural aging of skeletal muscle, and therefore our D-galactose-treated rats were suitable representatives of a premature aging model of skeletal muscle. However, skeletal muscle is one of the major organs responsible for metabolizing galactose via glycolysis, the pentose phosphate pathway, and reduction to galactitol [40]. Hence, measuring the levels of galactitol and metabolites involved in the pentose phosphate pathway should be performed to further clarify whether D-galactose is truly appropriate for inducing a natural aging-mimicked model in skeletal muscle. Interestingly, exercise and caloric restriction failed to alter the level of p21 and SA-β-gal proteins in skeletal muscle, suggesting that these interventions could not attenuate cell cycle arrest in skeletal muscle in the aging condition. However, prior studies in young rodents demonstrated that exercise and caloric restriction promoted genomic stability, which involved cell cycle regulation [41,42]. All of these results suggest that the genetic effect of these interventions is age dependent.

The maintenance of skeletal muscle protein via amino acid metabolism and growth factor regulation is a crucial factor for the maintenance of its mechanical function [43,44]. We observed that exercise and caloric restriction equally improved growth factor signaling and amino acid metabolism in the skeletal muscle of aged rats, all of which contributed to an equal improvement in myogenesis in the aging condition. Nevertheless, a balance between protein synthesis and protein degradation, or “protein turnover”, is also a key indicator to determine myogenesis [45]. Therefore, a future study evaluating protein turnover in skeletal muscle should be performed.

In the sedentary state, we found that exercise was superior to caloric restriction in terms of improving fatty acid oxidation in skeletal muscle. This was consistent with previous results from studies in obese rats [46] and obese individuals [20], suggesting that the effects of exercise and caloric restriction on skeletal muscle fatty acid oxidation at rest were similar between obese and aging conditions. An improvement in fatty acid oxidation consequently resulted in improved oxidative phosphorylation and ATP production in D-galactose-treated rats with exercise [47]. On the other hand, caloric restriction was more effective than exercise in improving fatty acid uptake into skeletal muscle mitochondria for fatty acid oxidation. All of these findings suggested that a combination of exercise and caloric restriction should be superior to either exercise or caloric restriction alone in terms of improving skeletal muscle fatty acid oxidation in the aging condition.

It is well known that an improvement in fatty acid oxidation in skeletal muscle leads to a decrease in skeletal muscle fat accumulation and, hence, improved skeletal muscle insulin sensitivity [48]. Surprisingly, our D-galactose-treated rats with exercise did not exhibit an improvement in insulin signaling and glycolysis in skeletal muscle in the sedentary state, despite an improvement in fatty acid oxidation in skeletal muscle. This could be explained by an increase in the ROS level of the skeletal muscle following exercise. In fact, it has been suggested that increased oxidative phosphorylation results in increased ROS production [49]. Without an adequate antioxidative capacity, increased ROS levels lead to increased oxidative stress, which is a major contributor to insulin resistance [50,51]. The effect of chronic exercise on increased oxidative stress in skeletal muscle has been widely reported [52,53], and therefore treatment with antioxidants during exercise has been recommended [52,53,54]. Unlike our findings, a previous study showed that exercise and caloric restriction alleviated obesity-induced skeletal muscle insulin resistance [16,17,20], suggesting that the benefit of exercise and caloric restriction on the improvement of skeletal muscle insulin sensitivity is highly dependent on obesity status. Moreover, it was observed that chronic exercise in young humans could counteract age-induced skeletal muscle insulin resistance [55]. This finding suggests that early lifelong exercise has greater potential than late exercise in terms of ameliorating aging-induced skeletal muscle insulin resistance in the sedentary state.

During physical activity, we observed that caloric restriction restored the whole-body oxidation rate of fatty acids, whereas exercise could increase this parameter in D-galactose-induced aged rats when compared to that of young adults. As skeletal muscle is a major fuel source during exercise [56,57], all of these results indicated that caloric restriction restored skeletal muscle fatty acid oxidation while exercise increased skeletal muscle fatty acid oxidation during active physical activity in D-galactose-induced aged rats. It also indicates that exercise and caloric restriction could restore whole-body carbohydrate oxidation rate during physical activity, showing that both interventions ameliorated skeletal muscle insulin resistance during active physical activity in D-galactose-induced aged rats. Indeed, the results suggested that an exercise-induced increase in skeletal muscle fatty acid oxidation was somewhat effective in causing an improvement in skeletal muscle insulin sensitivity during physical activity, despite increased ROS production. In addition, the restoration of skeletal muscle fatty acid oxidation by caloric restriction resulted in a restoration of skeletal muscle insulin sensitivity during active physical activity. All of these results also suggested that the effect of exercise and caloric restriction on an improvement in skeletal muscle metabolism in the aging condition is more apparent during physical activity than during the resting state.

Although we did not observe any improvement in skeletal muscle insulin sensitivity in the resting state following exercise and caloric restriction, blood metabolic profiles showed that exercise restored systemic insulin sensitivity while caloric restriction increased systemic insulin sensitivity in D-galactose-induced aged rats, in comparison to that of young adults. Liver and adipose tissue also determine systemic insulin sensitivity [58]; therefore, it is likely that the benefit of exercise and caloric restriction observed in our study was mediated by an improvement in hepatic and adipocyte insulin sensitivity. To support this explanation, further related studies in the liver and adipose tissue are needed.

Our results revealed that caloric restriction improved the antioxidative capacity in skeletal muscle of D-galactose-induced aged rats, as a reduction of skeletal muscle ROS level was exhibited in our D-galactose-treated rats with caloric restriction. These findings were consistent with several previous studies [59,60,61]. As previously mentioned, our findings suggested that exercise failed to attenuate aging-induced skeletal muscle insulin resistance in the sedentary state due to exercise-induced increased oxidative stress. Therefore, it is likely that a combination of exercise and caloric restriction can effectively improve skeletal muscle insulin sensitivity at the resting state in the aging condition via exercise-induced improved fatty acid oxidation together with caloric restriction-induced decreased oxidative stress.

To determine the mechanical function of skeletal muscle, we evaluated locomotor activity. The results showed that exercise was superior to caloric restriction in improving these parameters. It has been widely acknowledged that fatty acid oxidation in skeletal muscle is a crucial factor that determines the level of physical endurance of skeletal muscle [62]. Hence, it is not surprising that our results regarding locomotor activity were consistent with those of skeletal muscle fatty acid oxidation.

Interestingly, we found that the impairment of some parameters was more severe in naturally aged rats than those of D-galactose-treated rats, including plasma triglyceride level, plasma LDL cholesterol, and whole-body fatty acid and carbohydrate oxidation during physical activity. All these parameters are highly associated with obesity [63,64]; therefore, all of these findings could be explained by higher body weight and visceral fat weight in naturally aged rats when compared to those of D-galactose-treated rats, regardless of food intake. To identify the mechanism mediating the higher adiposity in naturally aged rats, testosterone deficiency was evaluated [65]. The results revealed that naturally aged rats had lower levels of serum testosterone than those of young adults; however, this change in level was not found in D-galactose-treated rats (Supplementary Figure S1). All of these findings indicated that D-galactose failed to induce male reproductive aging and consequently failed to completely mimic the natural aging of some parameters. However, significant alterations in plasma triglyceride level, plasma LDL cholesterol, and whole-body fatty acid and carbohydrate oxidation during physical activity were evident in D-galactose-treated rats when compared with those of young adults. These suggest that D-galactose administration is still an effective method to induce premature aging.

In summary, our results indicated that exercise and caloric restriction exert beneficial effects on the metabolism and mechanical function of skeletal muscle. These benefits did differ between these two interventions. Exercise was shown to be superior to caloric restriction in terms of improving skeletal muscle fatty acid oxidation and locomotor activity, whereas caloric restriction is superior to exercise in terms of decreasing oxidative stress and increasing antioxidative capacity in skeletal muscle. However, these findings may not be applicable to females since the skeletal muscle structure and skeletal muscle metabolism of males and females are different [66,67,68], and sex hormones play crucial roles in skeletal muscle metabolic and mechanical functions [69,70,71]. Hence, future studies investigating the beneficial effects of exercise and caloric restriction on the amelioration of skeletal muscle aging in females are needed.

## Data Availability

The data that support the findings of this study are available from the corresponding author upon reasonable request.

## Supplementary Materials

The following supporting information can be downloaded at: https://www.mdpi.com/article/10.3390/nu15235004/s1, Table S1: The levels of skeletal muscle metabolomes; Figure S1: Serum testosterone level.

## References

[1] Knickman J.R., Snell E.K. (2002). The 2030 problem: Caring for aging baby boomers. Health Serv. Res..

[2] Brody J.A., Grant M.D. (2001). Age-associated diseases and conditions: Implications for decreasing late life morbidity. Aging.

[3] McCormick R., Vasilaki A. (2018). Age-related changes in skeletal muscle: Changes to life-style as a therapy. Biogerontology.

[4] Frontera W.R., Ochala J. (2015). Skeletal muscle: A brief review of structure and function. Calcif. Tissue Int..

[5] Mitchell W.K., Wilkinson D.J., Phillips B.E., Lund J.N., Smith K., Atherton P.J. (2016). Human Skeletal Muscle Protein Metabolism Responses to Amino Acid Nutrition. Adv. Nutr..

[6] Blum J., Epstein R., Watts S., Thalacker-Mercer A. (2021). Importance of Nutrient Availability and Metabolism for Skeletal Muscle Regeneration. Front. Physiol..

[7] Thyfault J.P., Kraus R.M., Hickner R.C., Howell A.W., Wolfe R.R., Dohm G.L. (2004). Impaired plasma fatty acid oxidation in extremely obese women. Am. J. Physiol. Endocrinol. Metab..

[8] Stump C.S., Henriksen E.J., Wei Y., Sowers J.R. (2006). The metabolic syndrome: Role of skeletal muscle metabolism. Ann. Med..

[9] Rahman F.A., Quadrilatero J. (2021). Mitochondrial network remodeling: An important feature of myogenesis and skeletal muscle regeneration. Cell. Mol. Life Sci..

[10] Bentzinger C.F., Wang Y.X., Rudnicki M.A. (2012). Building muscle: Molecular regulation of myogenesis. Cold Spring Harb. Perspect. Biol..

[11] Husmann I., Soulet L., Gautron J., Martelly I., Barritault D. (1996). Growth factors in skeletal muscle regeneration. Cytokine Growth Factor Rev..

[12] Arsic N., Zacchigna S., Zentilin L., Ramirez-Correa G., Pattarini L., Salvi A., Sinagra G., Giacca M. (2004). Vascular endothelial growth factor stimulates skeletal muscle regeneration in vivo. Mol. Ther..

[13] Glass D.J. (2010). PI3 kinase regulation of skeletal muscle hypertrophy and atrophy. Curr. Top. Microbiol. Immunol..

[14] Thyfault J.P., Bergouignan A. (2020). Exercise and metabolic health: Beyond skeletal muscle. Diabetologia.

[15] Pérez-Rodríguez M., Huertas J.R., Villalba J.M., Casuso R.A. (2022). Mitochondrial adaptations to calorie restriction and bariatric surgery in human skeletal muscle: A systematic review with meta-analysis. Metabolism.

[16] Amati F., Dubé J.J., Coen P.M., Stefanovic-Racic M., Toledo F.G., Goodpaster B.H. (2009). Physical inactivity and obesity underlie the insulin resistance of aging. Diabetes Care.

[17] Amati F., Pennant M., Azuma K., Dubé J.J., Toledo F.G., Rossi A.P., Kelley D.E., Goodpaster B.H. (2012). Lower thigh subcutaneous and higher visceral abdominal adipose tissue content both contribute to insulin resistance. Obesity.

[18] Safdar A., Hamadeh M.J., Kaczor J.J., Raha S., Debeer J., Tarnopolsky M.A. (2010). Aberrant mitochondrial homeostasis in the skeletal muscle of sedentary older adults. PLoS ONE.

[19] Lanza I.R., Short D.K., Short K.R., Raghavakaimal S., Basu R., Joyner M.J., McConnell J.P., Nair K.S. (2008). Endurance exercise as a countermeasure for aging. Diabetes.

[20] Menshikova E.V., Ritov V.B., Dube J.J., Amati F., Stefanovic-Racic M., Toledo F.G.S., Coen P.M., Goodpaster B.H. (2017). Calorie Restriction-induced Weight Loss and Exercise Have Differential Effects on Skeletal Muscle Mitochondria Despite Similar Effects on Insulin Sensitivity. J. Gerontol. A Biol. Sci. Med. Sci..

[21] Leenders M., Verdijk L.B., van der Hoeven L., van Kranenburg J., Nilwik R., van Loon L.J. (2013). Elderly men and women benefit equally from prolonged resistance-type exercise training. J. Gerontol. A Biol. Sci. Med. Sci..

[22] Bua E., McKiernan S.H., Aiken J.M. (2004). Calorie restriction limits the generation but not the progression of mitochondrial abnormalities in aging skeletal muscle. FASEB J..

[23] Yu D., Tomasiewicz J.L., Yang S.E., Miller B.R., Wakai M.H., Sherman D.S., Cummings N.E., Baar E.L., Brinkman J.A., Syed F.A. (2019). Calorie-Restriction-Induced Insulin Sensitivity Is Mediated by Adipose mTORC2 and Not Required for Lifespan Extension. Cell Rep..

[24] Shwe T., Pratchayasakul W., Chattipakorn N., Chattipakorn S.C. (2018). Role of D-galactose-induced brain aging and its potential used for therapeutic interventions. Exp. Gerontol..

[25] Bo-Htay C., Palee S., Apaijai N., Chattipakorn S.C., Chattipakorn N. (2018). Effects of d-galactose-induced ageing on the heart and its potential interventions. J. Cell. Mol. Med..

[26] Pantiya P., Thonusin C., Ongnok B., Chunchai T., Kongkaew A., Nawara W., Arunsak B., Chattipakorn N., Chattipakorn S.C. (2023). Chronic D-galactose administration induces natural aging characteristics, in rat’s brain and heart. Toxicology.

[27] Thonusin C., Pantiya P., Sumneang N., Chunchai T., Nawara W., Arunsak B., Siri-Angkul N., Sriwichaiin S., Chattipakorn S.C., Chattipakorn N. (2022). Effectiveness of high cardiorespiratory fitness in cardiometabolic protection in prediabetic rats. Mol. Med..

[28] Wang R., Tian H., Guo D., Tian Q., Yao T., Kong X. (2020). Impacts of exercise intervention on various diseases in rats. J. Sport Health Sci..

[29] Lee J., Cho J.Y., Kim W.K. (2014). Anti-inflammation effect of Exercise and Korean red ginseng in aging model rats with diet-induced atherosclerosis. Nutr. Res. Pract..

[30] Friedewald W.T., Levy R.I., Fredrickson D.S. (1972). Estimation of the concentration of low-density lipoprotein cholesterol in plasma, without use of the preparative ultracentrifuge. Clin. Chem..

[31] Overmyer K.A., Evans C.R., Qi N.R., Minogue C.E., Carson J.J., Chermside-Scabbo C.J., Koch L.G., Britton S.L., Pagliarini D.J., Coon J.J. (2015). Maximal oxidative capacity during exercise is associated with skeletal muscle fuel selection and dynamic changes in mitochondrial protein acetylation. Cell Metab..

[32] Farinatti P., Castinheiras Neto A.G., Amorim P.R. (2016). Oxygen Consumption and Substrate Utilization During and After Resistance Exercises Performed with Different Muscle Mass. Int. J. Exerc. Sci..

[33] Gonzalez J.T., Green B.P., Campbell M.D., Rumbold P.L., Stevenson E.J. (2014). The influence of calcium supplementation on substrate metabolism during exercise in humans: A randomized controlled trial. Eur. J. Clin. Nutr..

[34] Thonusin C., Nawara W., Khuanjing T., Prathumsup N., Arinno A., Ongnok B., Arunsak B., Sriwichaiin S., Chattipakorn S.C., Chattipakorn N. (2022). Blood metabolomes as non-invasive biomarkers and targets of metabolic interventions for doxorubicin and trastuzumab-induced cardiotoxicity. Arch. Toxicol..

[35] Thonusin C., IglayReger H.B., Soni T., Rothberg A.E., Burant C.F., Evans C.R. (2017). Evaluation of intensity drift correction strategies using MetaboDrift, a normalization tool for multi-batch metabolomics data. J. Chromatogr. A.

[36] Thonusin C., Apaijai N., Jaiwongkam T., Kerdphoo S., Arunsak B., Amput P., Palee S., Pratchayasakul W., Chattipakorn N., Chattipakorn S.C. (2019). The comparative effects of high dose atorvastatin and proprotein convertase subtilisin/kexin type 9 inhibitor on the mitochondria of oxidative muscle fibers in obese-insulin resistant female rats. Toxicol. Appl. Pharmacol..

[37] Thonusin C., Pantiya P., Jaiwongkam T., Kerdphoo S., Arunsak B., Amput P., Palee S., Pratchayasakul W., Chattipakorn N., Chattipakorn S.C. (2020). A proprotein convertase subtilisin/kexin type 9 inhibitor provides comparable efficacy with lower detriment than statins on mitochondria of oxidative muscle of obese estrogen-deprived rats. Menopause.

[38] Papismadov N., Gal H., Krizhanovsky V. (2017). The anti-aging promise of p21. Cell Cycle.

[39] Lee B.Y., Han J.A., Im J.S., Morrone A., Johung K., Goodwin E.C., Kleijer W.J., DiMaio D., Hwang E.S. (2006). Senescence-associated beta-galactosidase is lysosomal beta-galactosidase. Aging Cell.

[40] Conte F., van Buuringen N., Voermans N.C., Lefeber D.J. (2021). Galactose in human metabolism, glycosylation and congenital metabolic diseases: Time for a closer look. Biochim. Biophys. Acta Gen. Subj..

[41] Cabelof D.C., Yanamadala S., Raffoul J.J., Guo Z., Soofi A., Heydari A.R. (2003). Caloric restriction promotes genomic stability by induction of base excision repair and reversal of its age-related decline. DNA Repair.

[42] Werner C., Hanhoun M., Widmann T., Kazakov A., Semenov A., Pöss J., Bauersachs J., Thum T., Pfreundschuh M., Müller P. (2008). Effects of physical exercise on myocardial telomere-regulating proteins, survival pathways, and apoptosis. J. Am. Coll. Cardiol..

[43] Kamei Y., Hatazawa Y., Uchitomi R., Yoshimura R., Miura S. (2020). Regulation of Skeletal Muscle Function by Amino Acids. Nutrients.

[44] Syverud B.C., VanDusen K.W., Larkin L.M. (2016). Growth Factors for Skeletal Muscle Tissue Engineering. Cells Tissues Organs.

[45] Ferreira R.P., Duarte J.A. (2023). Protein Turnover in Skeletal Muscle: Looking at Molecular Regulation towards an Active Lifestyle. Int. J. Sports Med..

[46] Gopalan V., Michael N., Ishino S., Lee S.S., Yang A.Y., Bhanu Prakash K.N., Yaligar J., Sadananthan S.A., Kaneko M., Zhou Z. (2016). Effect of Exercise and Calorie Restriction on Tissue Acylcarnitines, Tissue Desaturase Indices, and Fat Accumulation in Diet-Induced Obese Rats. Sci. Rep..

[47] Nsiah-Sefaa A., McKenzie M. (2016). Combined defects in oxidative phosphorylation and fatty acid β-oxidation in mitochondrial disease. Biosci. Rep..

[48] Hamrick M.W., McGee-Lawrence M.E., Frechette D.M. (2016). Fatty Infiltration of Skeletal Muscle: Mechanisms and Comparisons with Bone Marrow Adiposity. Front. Endocrinol..

[49] Stojković B.M., Đorđević M. (2017). Interaction between mitochondrial and nuclear genomes: The role in life-history evolution. Biol. Serbica.

[50] Henriksen E.J., Diamond-Stanic M.K., Marchionne E.M. (2011). Oxidative stress and the etiology of insulin resistance and type 2 diabetes. Free Radic. Biol. Med..

[51] Birben E., Sahiner U.M., Sackesen C., Erzurum S., Kalayci O. (2012). Oxidative stress and antioxidant defense. World Allergy Organ. J..

[52] Thirupathi A., Pinho R.A., Chang Y.Z. (2020). Physical exercise: An inducer of positive oxidative stress in skeletal muscle aging. Life Sci..

[53] Taherkhani S., Valaei K., Arazi H., Suzuki K. (2021). An Overview of Physical Exercise and Antioxidant Supplementation Influences on Skeletal Muscle Oxidative Stress. Antioxidants.

[54] Antonioni A., Fantini C., Dimauro I., Caporossi D. (2019). Redox homeostasis in sport: Do athletes really need antioxidant support?. Res. Sports Med..

[55] Cartee G.D., Hepple R.T., Bamman M.M., Zierath J.R. (2016). Exercise Promotes Healthy Aging of Skeletal Muscle. Cell Metab..

[56] Hargreaves M., Spriet L.L. (2018). Exercise Metabolism: Fuels for the Fire. Cold Spring Harb. Perspect. Med..

[57] Hargreaves M., Spriet L.L. (2020). Skeletal muscle energy metabolism during exercise. Nat. Metab..

[58] Korenblat K.M., Fabbrini E., Mohammed B.S., Klein S. (2008). Liver, muscle, and adipose tissue insulin action is directly related to intrahepatic triglyceride content in obese subjects. Gastroenterology.

[59] Lanza I.R., Zabielski P., Klaus K.A., Morse D.M., Heppelmann C.J., Bergen H.R., Dasari S., Walrand S., Short K.R., Johnson M.L. (2012). Chronic caloric restriction preserves mitochondrial function in senescence without increasing mitochondrial biogenesis. Cell Metab..

[60] Feuers R.J., Weindruch R., Hart R.W. (1993). Caloric restriction, aging, and antioxidant enzymes. Mutat. Res..

[61] Gredilla R., Barja G. (2005). Minireview: The role of oxidative stress in relation to caloric restriction and longevity. Endocrinology.

[62] Kelley D.E. (2005). Skeletal muscle fat oxidation: Timing and flexibility are everything. J. Clin. Investig..

[63] Klop B., Elte J.W., Cabezas M.C. (2013). Dyslipidemia in obesity: Mechanisms and potential targets. Nutrients.

[64] Bell J.A., Reed M.A., Consitt L.A., Martin O.J., Haynie K.R., Hulver M.W., Muoio D.M., Dohm G.L. (2010). Lipid partitioning, incomplete fatty acid oxidation, and insulin signal transduction in primary human muscle cells: Effects of severe obesity, fatty acid incubation, and fatty acid translocase/CD36 overexpression. J. Clin. Endocrinol. Metab..

[65] Kelly D.M., Jones T.H. (2015). Testosterone and obesity. Obes. Rev. Off. J. Int. Assoc. Study Obes..

[66] You J.S., Barai P., Chen J. (2023). Sex differences in skeletal muscle size, function, and myosin heavy chain isoform expression during post-injury regeneration in mice. Physiol. Rep..

[67] Haizlip K.M., Harrison B.C., Leinwand L.A. (2015). Sex-based differences in skeletal muscle kinetics and fiber-type composition. Physiology.

[68] Lundsgaard A.M., Kiens B. (2014). Gender differences in skeletal muscle substrate metabolism–molecular mechanisms and insulin sensitivity. Front. Endocrinol..

[69] Alexander S.E., Pollock A.C., Lamon S. (2022). The effect of sex hormones on skeletal muscle adaptation in females. Eur. J. Sport Sci..

[70] Enns D.L., Tiidus P.M. (2010). The influence of estrogen on skeletal muscle: Sex matters. Sports Med..

[71] Herbst K.L., Bhasin S. (2004). Testosterone action on skeletal muscle. Curr. Opin. Clin. Nutr. Metab. Care.

