# Supplementary material for: Exercise and Caloric Restriction Exert Different Benefits on Skeletal Muscle Metabolism in Aging Condition
Source: Nutrients. 2023 Dec 3;15(23):5004. doi: 10.3390/nu15235004 (PMC10708263; doi:10.3390/nu15235004)

Supplementary Table S1: The levels of skeletal muscle metabolomes

Metabolomes	Young adult	Natural aging	D-gal	D-gal with EX	D-gal with CR
**Amino acids**					
Alanine (peak area/mg)	693071.87 ± 27836.05	689537.12 ± 73839.43	740866.21 ± 13470.01	698189.43± 50259.68	719349.48 ± 22088.64
Arginine (nmol/mg)	724.72 ± 47.69	1262.00 ± 175.55*	1255.21 ± 136.21*	504.41 ± 73.56†‡	1023.18 ± 133.34§
Asparagine (peak area/mg)	13435.81 ± 562.76	8182.03 ± 451.21*	7955.11 ± 378.68*	7009.79 ± 439.44*	11246.39 ± 397.95*†‡§
Aspartate (nmol/mg)	737.36 ± 66.94	458.34 ± 99.43*	469.05 ± 34.19*	497.69 ± 36.29*	509.09 ± 87.62*
Glutamate (nmol/mg)	432.69 ± 42.47	477.76 ± 71.36	425.91 ± 72.08	472.89 ± 13.43	414.83 ± 61.68
Glutamine (peak area/mg)	416546.37 ± 31869.25	558066.25 ± 54031.10*	526701.75 ± 29095.84*	399601.11 ± 29236.27†‡	552417.08 ± 18079.95*§
Glycine (nmol/mg)	312.59 ± 30.14	443.79 ± 37.04*	291.87 ± 17.95†	290.13 ± 14.10†	335.11 ± 26.78†
Histidine (µmol/mg)	3.86 ± 0.31	5.61 ± 0.08*	6.02 ± 0.46*	5.64 ± 0.59*	4.12 ± 0.29†‡§
Isoleucine and Leucine (nmol/mg)	418.61 ± 30.55	278.99 ± 20.28*	308.79 ± 14.50*	304.62 ± 26.33*	292.13 ± 25.05*
Lysine (µmol/mg)	2.38 ± 0.19	2.52 ± 0.55	2.34 ± 0.10	1.94 ± 0.37	2.49 ± 0.37
Methionine (nmol/mg)	228.38 ± 8.35	273.57 ± 18.68*	280.20 ± 7.07*	216.58 ± 18.56†‡	225.16 ± 9.04†‡
Phenylalanine (nmol/mg)	233.31 ± 10.04	287.59 ± 24.27*	283.60 ± 12.72*	209.48 ± 15.63†‡	207.34 ± 10.55†‡
Proline (nmol/mg)	750.33 ± 52.77	1244.35 ± 45.25*	1208.77 ± 36.66*	1007.39 ± 77.38*†‡	1000.73 ± 45.32*†‡
Serine (µmol/mg)	2.18 ± 0.31	4.05 ± 0.49*	3.81 ± 0.73*	2.31 ± 0.20	3.55 ± 0.34
Threonine (µmol/mg)	1.43 ± 0.13	2.32 ± 0.30*	2.15 ± 0.21*	1.50 ± 0.14†‡	1.90 ± 0.22
Tryptophan (peak area/mg)	446236.09 ± 22344.97	370167.99 ± 16763.93*	360416.70 ± 26965.90*	436962.22 ± 15768.61†‡	438380.32 ± 20135.02†‡
Tyrosine (nmol/mg)	569.52 ± 49.30	717.67 ± 47.80*	745.72 ± 17.18*	587.12 ± 49.56†‡	594.36 ± 19.88†‡
Valine (nmol/mg)	567.78 ± 38.64	803.59 ± 107.30*	726.75 ± 32.56*	662.34 ± 48.20†	550.50 ± 14.91†‡
**Glycolysis metabolites**					
Glucose (µmol/mg)	21.58 ± 1.54	23.99 ± 1.48	21.16 ± 1.24	20.25 ± 1.48	22.10 ± 0.82
Glucose-6-phosphate and Fructose-6-phosphate (µmol/mg)	29.78 ± 5.33	7.83 ± 0.77*	4.81 ± 1.47*	8.41 ± 0.43*	8.42 ± 3.54*
Fructose-1,6-bisphosphate (µmol/mg)	11.31 ± 0.44	7.95 ± 1.71*	7.41 ± 0.81*	7.44 ± 0.50*	7.72 ± 0.89*
Glyceraldehyde-3-phosphate (peak area/mg)	1149563.09 ± 210320.63	2262417.82 ± 133310.51*	2156384.85 ± 219666.05*	2223418.29 ± 127952.28*	2005588.07 ± 211867.22*
3-Phosphoglycerate and 2-Phosphoglycerate (µmol/mg)	1.01 ± 0.05	1.23 ± 0.05*	1.18 ± 0.03*	1.27 ± 0.08*	1.22 ± 0.05*
Lactate (µmol/mg)	1.12 ± 0.07	1.07 ± 0.14	1.20 ± 0.12	1.22 ± 0.22	1.19 ± 0.11

**Palmitic acid, oleic acid, and their long-chain acylcarnitine intermediates**					
Palmitic acid (nmol/mg)	163.62 ± 12.15	171.40 ± 40.17	140.61 ± 11.23	167.58 ± 8.93	131.06 ± 12.20
Oleic acid (µmol/mg)	6.31 ± 0.74	6.02 ± 1.50	6.27 ± 0.23	7.05 ± 0.39	6.64 ± 1.32
Palmitoylcarnitine (nmol/mg)	21.36 ± 2.29	3.85 ± 1.26*	5.92 ± 0.21*	2.33 ± 0.17*	12.80 ± 1.16*†‡§
Oleylcarnitine (peak area/mg)	26990358.69 ± 3486281.34	3449712.84 ± 888546.52*	5067101.91 ± 448705.78*	1352536.00 ± 11540.64*	11562165.25 ± 897114.26*†‡§
**Medium-chain acylcarnitine intermediates of palmitic acid and oleic acid**					
Hexanoylcarnitine (pmol/mg)	186.87 ± 16.24	182.53 ± 15.80	211.59 ± 61.90	75.70 ± 4.29*†‡	184.52 ± 17.66§
Octanoylcarnitine (pmol/mg)	253.44 ± 39.45	324.89 ± 79.14	269.99 ± 42.94	80.09 ± 7.56*†‡	253.65 ± 73.76§
Decanoyl carnitine (pmol/mg)	333.59 ± 61.71	280.22 ± 80.06	279.10 ± 74.79	69.00 ± 2.80*†‡	298.37 ± 51.43§
Lauroylcarnitine (pmol/mg)	172.94 ± 28.75	165.79 ± 44.11	154.99 ± 22.52	37.34 ± 5.73*†‡	170.22 ± 37.23§
Myristoylcarnitine (pmol/mg)	1101.11 ± 241.96	1121.44 ± 285.55	986.28 ± 201.77	233.67 ± 34.69*†‡	1029.34 ± 158.27
Dodecenoylcarnitine (peak area/mg)	46273.97 ± 8704.40	45028.82 ± 7009.65	42555.54 ± 9064.68	23782.81 ± 5348.33*†‡	43608.60 ± 5978.48§
Tetradecenoylcarnitine (peak area/mg)	138797.67 ± 30194.18	161178.77 ± 33 445.36	129747.16 ± 15062.47	18667.34 ± 628.54*†‡	143268.78 ± 18573.15§
**Adenosine diphosphate (ADP) and Adenosine triphosphate (ATP)**					
ADP (peak area/mg)	496158.52 ± 70121.76	509917.79 ± 63914.36	493816.35 ± 44607.58	478670.27 ± 45780.96	565403.39 ± 71936.61
ATP (µmol/mg)	7.81 ± 2.39	3.71 ± 0.38*	3.43 ± 0.38*	7.53 ± 0.53†‡	3.98 ± 0.42§

Data are reported as mean ± SEM. n = 6 per group. D-gal = D-galactose induced premature aging, D-gal with EX = D-gal receiving exercise, D-gal with CR = D-gal receiving caloric restriction. * $p < 0.05$ when compared to Young adult, † $p < 0.05$ when compared to Natural aging , ‡ $p < 0.05$ when compared to D-gal, § $p < 0.05$ when compared to D-gal with EX.

Supplementary Figure S1. Serum testosterone level

Data are reported as mean ± SEM. n=6 per group

D-gal = D-galactose-induced premature aging

* p <0.05 when compared to young adult, † p <0.05 when compared to natural aging

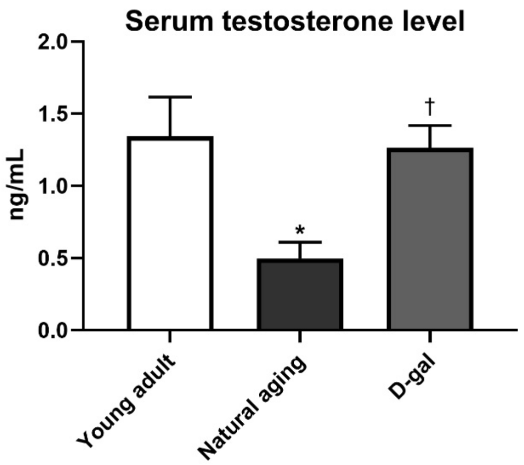

Supplement: Supplementary file 1 [file nutrients-15-05004-s001.zip › nutrients-2732977-supplementary.pdf]
